# Infantile hepatic hemangioendothelioma associated with pulmonary artery hypertension and cardiac insufficiency successfully treated with transcatheter arterial embolization and propranolol: A case report

**DOI:** 10.1097/MD.0000000000020728

**Published:** 2020-06-12

**Authors:** Liang Wang, Dan Song, Changhua Wu, Jing Li, Jie Yin, Lei Guo

**Affiliations:** a Department of Vascular Anomalies and Interventional Radiology, Ji’nan Children's Hospital, Jinan; b Department of Interventional Radiology, Beijing Children's Hospital of Capital Medical University, Beijing, China.

**Keywords:** cardiac insufficiency, embolization, hemangioendothelioma, propranolol, pulmonary artery hypertension

## Abstract

**Introduction:**

Infantile hepatic hemangioendothelioma is a type of benign hepatic tumor that occurs in infancy. Many hepatic tumors are diagnosed when screening is done for multiple cutaneous hemangiomas. Hepatic tumors are small multifocal lesions and are mostly asymptomatic. There have been many case reports of asymptomatic infantile hepatic hemangioendothelioma, but few of these have pointed out that hepatic hemangiomas can sometimes be life-threatening due to fatal hepatomegaly complications such as pulmonary artery hypertension or even congestive heart failure. At present, there are no standard protocols for treating infantile hepatic hemangioendothelioma, though most clinicians agree that treatment is unnecessary for multiple small hepatic hemangiomas in asymptomatic patients. Little is known about treatment for cases with life-threatening complications induced by infantile hepatic hemangioendothelioma as there are so few reported cases. Here, we report a special case with hepatomegaly, pulmonary artery hypertension, and cardiac insufficiency induced by infantile hepatic hemangioendothelioma.

**Patient concerns:**

We present a case with hepatomegaly, pulmonary artery hypertension, and cardiac insufficiency caused by infantile hepatic hemangioendothelioma.

**Diagnosis:**

Infantile hepatic hemangioendothelioma was diagnosed.

**Interventions:**

The patient underwent transcatheter arterial embolization and was given propranolol.

**Outcomes:**

The patient responded well to treatment with transcatheter arterial embolization and propranolol. The patient gained weight steadily, liver volume, and mass size have decreased considerably, echocardiography showed normal pulmonary artery pressure and ejection fraction, and we discovered no serious complications after 1 year of follow-up.

**Conclusion:**

Transcatheter arterial embolization combined with propranolol is an effective treatment for life-threatening infantile hepatic hemangioendothelioma.

## 1 Introduction

Infantile hepatic hemangioendothelioma (IHH) is the most common benign hepatic tumor of infancy with an incidence of approximately 1 in 20,000^[1]^; IHH has a slight female preponderance.^[2–3]^ Hepatic hemangiomas can manifest in a spectrum of presentations ranging from natural regression without symptoms to life-threatening complications. Transcatheter arterial embolization (TAE) is an effective treatment for reducing shunts and counteracting cardiac failure for hepatic hemangiomas.^[4]^ In this report, we present a case with hepatomegaly, pulmonary artery hypertension (PAH) and cardiac insufficiency caused by infantile hepatic hemangioendothelioma; the patient's symptoms was well controlled with transcatheter arterial embolization and propranolol.

## 2 Case presentation

A 4-month-old male infant, after an uncomplicated pregnancy and delivery, weighed 3000 g at birth; there were no complications during pregnancy and no family history. The infant had symptoms of mild respiratory distress for 2 months, without fever or cough. He was diagnosed with patent foramen ovale by echocardiography at the primary hospital and received no treatments. Later, the symptoms were aggravated, and his parents sought care, again at the primary hospital, 9 days later. Ultrasonography of the abdomen showed features suggestive of hemangioendothelioma of liver. The hospital suggested that to the infant should be transferred to our hospital for treatment.

Examination revealed an irritable infant with cough, fever, breathlessness and cyanosis; the infant weighed 6.8 kg and was in respiratory distress with a relative ratio of approximately 130/min. The infant had multiple skin hemangiomas of varying sizes on the head, neck, right index finger, and right shank. His physical examination was remarkable for bilateral wheezy phlegm in both lung bases. The physical examination showed enlarged cardiac dullness, but there were no significant heart murmurs. Abdominal distention was noted. The liver was soft and located 8 cm below the costal margin at the right mid-clavicular line.

Echocardiography showed right atrial and right ventricular dilatation with a thickened right ventricular anterior wall and interventricular septum. The normal structure of the cardiac chamber and continuous atrioventricular septum were observed. The infant was diagnosed as having severe pulmonary artery hypertension with mild tricuspid regurgitation and trivial mitral regurgitation. Ultrasonography of the abdomen showed densely hypodense lesions, increased volume of liver lobes, with a clear boundary and a maximum diameter of 4.1 cm. The liver lobes had a heterogeneous internal echo and enhanced peripheral echo. Visible blood vessels were observed in the hypodense lesions. There were only a few normal liver parenchymas.

**Figure 1:**
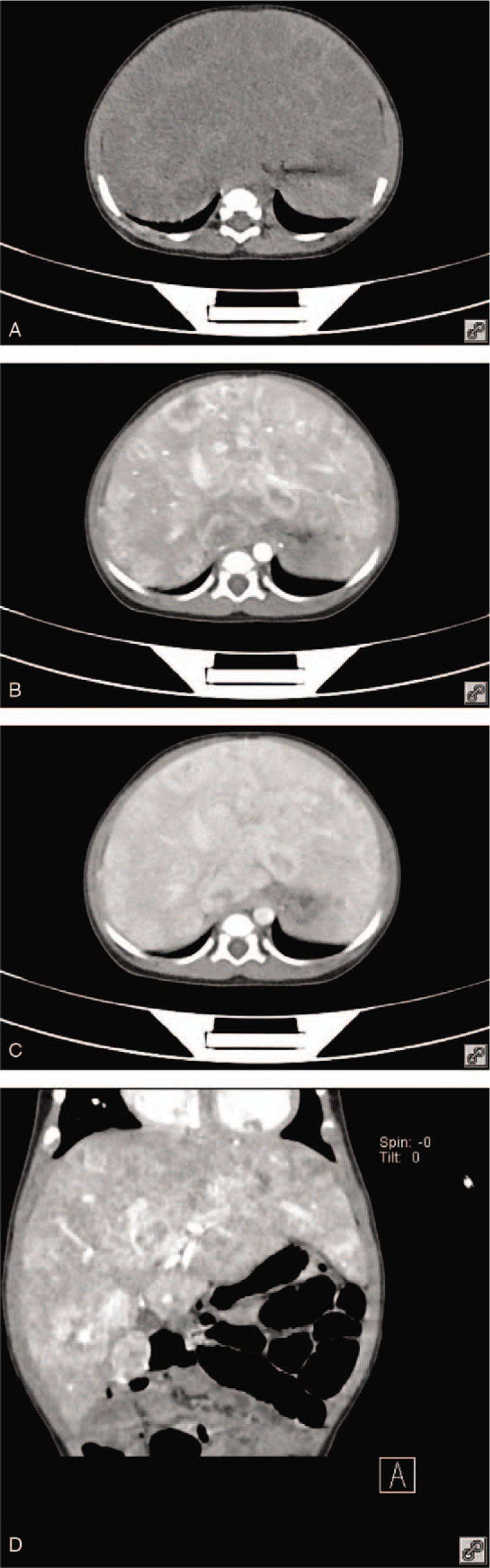
Computed tomography scan of the abdomen showed an enlarged, irregularly shaped liver with multiple low-density intrahepatic masses which present as enhance centripetally.

A computed tomography (CT) scan of the abdomen showed an enlarged, irregularly shaped liver with multiple low-density intrahepatic masses, and its CT value was approximately 43 HU. Contrast-enhanced abdomen CT showed significant enhancement in the periphery of tumor, uneven enhancement in the center part of tumor during the arterial phase, centripetal enhancement in portal vein phase and even enhancement in late phase of tumor, which was more enhanced than normal liver. The lesions were more uniformly strengthened, and their degree of strengthening was higher than that of normal liver tissue in the delayed period (Fig. 1).

The laboratory examinations showed normal values for creatine kinase, creatine kinase-MB, alanine aminotransferase, aspartate aminotransferase, unconjugated bilirubin, serum total bilirubin, conjugated bilirubin, creatinine, serum urea, and coagulant activity. However, thyroid-stimulating hormone (TSH) was 19.5 uIU/mL (NL = 1.36–8.8 uIU/mL), free triiodothyronine (fT3) was 3.21 pmol/L (NL = 4.5–10.5 pmol/L), and free thyroxine was normal. Meanwhile, alpha-fetoprotein was 15434.76 Ug/L (NL = 0–25 Ug/L).

After we discussed the patient's condition, the infant was diagnosed with hepatomegaly, pulmonary artery hypertension, cardiac insufficiency, pneumonia, hepatic hemangioendothelioma, hypothyroidism, and multiple hemangiomas. Then, PAH and cardiac insufficiency were managed with fluid restriction, oxygen, diuretics, and inotropic support. In addition, the pneumonia was managed with antibiotics. Because fT3 was mildly decreased, we suggested that the infant should be regularly seen for endocrinology consultation. Meanwhile, propranolol was given at a dosage of 1 mg/kg every 12 hours.

Eight days after admission, the patient underwent transcatheter arterial embolization under general anesthesia. Unfortunately, because the diagnosis was relatively clear and the condition of the infant was worsening, his parents agreed to allow the patient to undergo TAE and refused a digital subtraction angiography-guided percutaneous biopsy after we informed them of the clinical risk. In addition, the TAE was approved by the ethics committee of Ji’nan Children's Hospital. The perineum was disinfected and draped with the patient in the supine position. The femoral artery was punctured by the Seldinger technique, and 100 IU/kg of heparin was administered to avoid thrombosis. The celiac artery was catheterized with a 4-F PIG angiographic catheter (Cordis, MI) under X-ray guidance. We observed that the blood supply to the IHH was derived from the proper hepatic artery branches, including the left hepatic artery and right hepatic artery, by digital subtraction angiography. The celiac artery was catheterized again with a 4-F Cobra guide catheter (Terumo, Tokyo, Japan) under X-ray guidance. Then, the 2.6 F microcatheter (Asahi, Nagoya, Japan) was used for superselective catheterization of the feeding artery. During the procedure, a pingyangmycin-lipiodol emulsion was injected slowly through the catheter until the periphery of the hemangioma was completely surrounded. Gelatin sponge particle (350–560 μm) embolization of the feeding artery was performed if the blood supply artery was faster, as shown by angiography. Selective celiac arteriography was performed once again if necessary to judge the degree of embolism of the supplying arteries. The injection was stopped when a small branch of the portal vein around the tumor was developed or the total volume was administered. At the conclusion of the embolization, the microcatheter was withdrawn, and the sheath was removed. Hemostasis of the femoral artery was then achieved by manual compression for 10 to 15 minutes.

**Figure 2:**
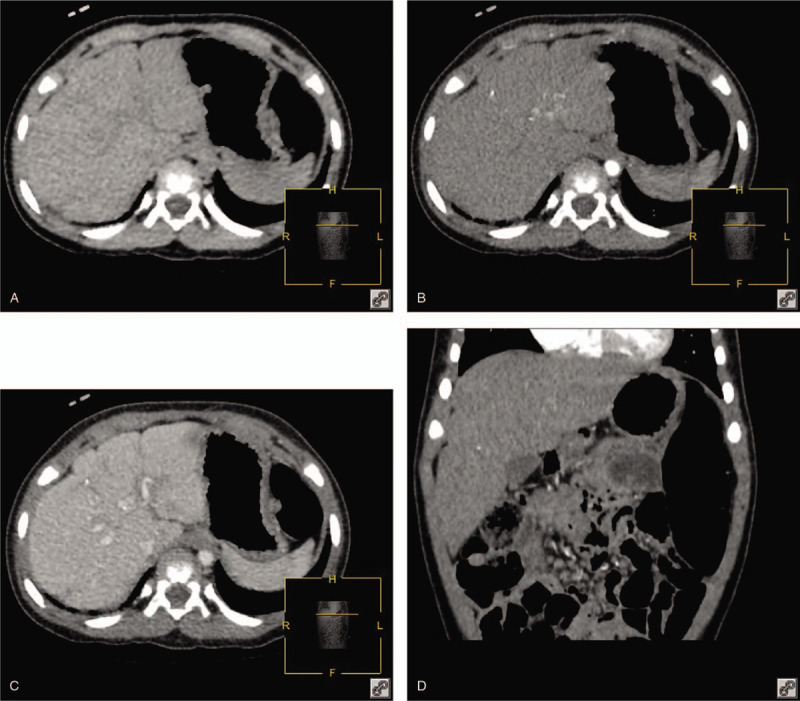
One year later, computed tomography scan of the abdomen showed the liver volume and size of the lesions had decreased considerably.

Postoperatively, meticulous nursing care was given to the patient, and symptomatic relief and supportive treatment were continued. Thirty-five days after admission, the PAH decreased, the symptoms of cardiac insufficiency were alleviated, and the severe pneumonia was cured. On the third month after the TAE, the abdominal distention was alleviated. The liver was soft and located 2 cm below the costal margin at the right mid-clavicular line. A CT scan of the abdomen still showed an irregularly shaped liver with multiple significantly strengthened intrahepatic masses, and the size of the largest mass was 1.6 cm. Echocardiography showed normal pulmonary artery pressure and EF. The patient underwent a second TAE under general anesthesia. There were no serious complications after the operation. At the 1-year follow-up, the infant had steadily gained weight, the liver volume and size of the mass decreased considerably (Fig. 2), and the hypothyroidism returned to normal.

## 3 Discussion

Clinical manifestations of IHH are mostly manifested as an abdominal mass in the first 6 months of life and may be accompanied by a cutaneous hemangioma.^[5]^ Many cases of IHH remain asymptomatic, and they are diagnosed by occasional ultrasound of the abdomen.^[6]^ However, sometimes arteriovenous shunts within IHH could generate fatal complications, including PAH and even congestive heart failure.^[7]^ The development of congestive heart failure may depend on decreased contraction and increased cardiac output,^[5]^ and multifocal and diffuse IHHs may be more prone to be detected. The high-output heart failure is mainly due to lower peripheral vascular resistance; because of large arteriovenous shunts, the perfusion of the vascular bed requires more blood volume and cardiac output.^[8]^ In addition, the high-output heart failure might also be related to the negative effect of hypothyroidism on cardiac functions.^[5]^ Meanwhile, changes in hemodynamics make patients vulnerable to severe pneumonia.^[4]^ A review of the patients’ symptoms did not reveal significant findings two months prior, and echocardiography was normal at the early stage of respiratory distress. When the symptoms were aggravated, ultrasonography and CT of the abdomen showed an enlarged, irregularly shaped liver with multiple densely intrahepatic masses, and echocardiography showed PAH and cardiac insufficiency. Meanwhile, the patient had developed pneumonia and hypothyroidism. Among these manifestations, the hypothyroidism might be related to the production of type 3 iodothyronine deiodinase enzyme.^[9]^ It has been reported that high-output heart failure developed in 30% of patients, and hypothyroidism developed in 22% of patients diagnosed with hepatic hemangioma in infancy.^[10]^ S et al^[5]^ reported that the development of hypothyroidism may increase morbidity and mortality, and thyroxine treatment was given at a dose higher than the routine dose to suppress TSH. Because fT3 had mildly decreased, although TSH increased markedly, we suggested that the infant should receive regular endocrinology consultation as an alternative to high dose L-thyroxin. Currently, the patients’ thyroid function has returned to normal.

The common options for treating hepatic hemangiomas include observation, pharmacologic therapy, embolization and surgical resection. However, there are no standard protocols for treating IHH. Treatment is unnecessary for multiple small hepatic hemangiomas in an asymptomatic patient. Cautious observation and monitoring are indicated, as long as there is no hepatomegaly, signs of incipient cardiac failure, or imaging findings that demonstrate fast-flow macrovascular shunting. Because most cases of IHH are asymptomatic with spontaneous regression, cases with heart failure experience a high mortality rate.^[11]^ Otherwise, patients should be given drug therapy using the same protocols as for problematic cutaneous hemangiomas, and the effectiveness of IHHs treatment with propranolol has been reported.^[12–14]^ In addition, propranolol has been the first-line treatment for rapidly proliferating hemangiomas since 2008 because of its high efficacy and minimal adverse reactions. The regimen is propranolol 1 to 3 mg/kg/d, which is divided into 2 to 3 doses. Usually, blood pressure and cardiac rate are monitored at the beginning of treatment, and the dose of propranolol is increased in proportion to the infant's weight gain.

Embolization may be considered in critically ill infants who require mechanical ventilation or vasopressor therapy. TAE is an effective treatment for reducing shunting for hemangiomas. It can be considered when IHH is complicated by arteriovenous malformations and high-output congestive heart failure,^[15]^ and TAE is considered an effective treatment for reducing shunts and counteracting cardiac failure for hepatic hemangiomas.^[4]^ Prior to embolization, hepatic lesions should be carefully studied by aortography focused on systemic vascular supply, and by superior mesenteric arteriography to demonstrate the portal venous phase. Additionally, staged procedures are prudent to minimize the risk of hepatic necrosis and death.

Some postoperative complications deserve our attention. The most common complication is fever. We recommend giving sufficient continuous rehydration therapy to decrease the treatment toxicity after the operation. According to our past experience,^[16]^ approximately half of the patients had low-grade fever after TAE and were healed with symptomatic treatment. Meanwhile, nausea, vomiting, and abdominal distention occurred in a few patients, and there were few patients who showed alanine aminotransferase and aspartate aminotransferase increased after TAE and had no serious complications. We performed TAE on a 4-month-old male infant suffering from multifocal IHH that caused massive hepatomegaly, PAH, cardiac insufficiency, severe pneumonia and hypothyroidism. Postoperatively, the pulmonary artery hypertension decreased immediately, and the symptoms of cardiac insufficiency were alleviated. After standard treatment, severe pneumonia was cured. In addition, at the 1-year follow-up, the infant was gaining weight steadily, the liver volume and size of the mass had decreased considerably, echocardiography showed normal pulmonary artery pressure and ejection fraction, and there were no serious complications.

In our experience, cautious observation is important for the asymptomatic patient with infantile hepatic hemangioendothelioma. Although most cases of IHH are asymptomatic with spontaneous regression, it is emphasized that early aggressive treatment in symptomatic IHH is important, and propranolol or/and transcatheter arterial embolization is an effective treatment for IHH with life-threatening complications.

## Author contributions

**Conceptualization:** LG.

**Data curation:** Liang Wang, Jing Li.

**Formal analysis:** Changhua Wu.

**Methodology:** Jie Yin.

**Project administration:** Dan Song.

**Supervision:** ChW.

**Validation:** DS.

**Visualization:** Lei Guo.

**Writing – original draft:** Liang Wang.
